# CXCL12–CXCR4 signalling axis confers gemcitabine resistance to pancreatic cancer cells: a novel target for therapy

**DOI:** 10.1038/sj.bjc.6605968

**Published:** 2010-11-02

**Authors:** S Singh, S K Srivastava, A Bhardwaj, L B Owen, A P Singh

**Affiliations:** 1 Department of Oncologic Sciences, Mitchell Cancer Institute, University of South Alabama, 1660 Springhill Avenue, Mobile, Alabama 36604-1405, USA; 2 Department of Biochemistry and Molecular Biology, University of South Alabama, Mobile, Alabama 36688, USA

**Keywords:** CXCR4, CXCL12, pancreatic cancer, drug resistance, therapeutic target

## Abstract

**Background:**

Pancreatic cancer cells are highly resistant to drug therapy; however, underlying causes remain largely unknown. We hypothesised that the activation of CXCL12–CXCR4 signalling confers drug resistance to pancreatic cancer cells by potentiating survival. CXCR4 is overexpressed in precancerous/malignant pancreatic lesions and cancer stem cells, and implicated in its pathogenesis.

**Methods:**

Effect of CXCR4 activation by CXCL12 on restricting the gemcitabine-induced cytotoxicity and stimulating the survival signalling was examined in pancreatic cancer cells by MTT, DNA laddering, caspase activity, immunoblot, and promoter-reporter assays. Subsequently, we examined the effect of CXCR4 antagonist, AMD3100, in abrogating the rescue effect of activated CXCL12–CXCR4 signalling.

**Results:**

The pancreatic cancer cells treated with gemcitabine exhibited reduced cytotoxicity in the presence of CXCL12 as compared with the cells treated with drug alone. CXCL12 induced the activation of FAK, ERK, and Akt signalling pathways, enhanced transcriptional activities of *β*-catenin and NF-*κ*B, and expression of survival proteins. AMD3100 arrested the CXCL12-induced pancreatic cancer cell growth and drug resistance.

**Conclusion:**

Our findings demonstrate, for the first time, a role of CXCL12–CXCR4 signalling axis in conferring drug resistance to pancreatic cancer cells and suggest that it could serve as a novel therapeutic target for pancreatic cancer therapy, alone and in combination with the cytotoxic drug.

Pancreatic cancer is a highly lethal malignancy with an extremely poor prognosis. The overall median survival after diagnosis is 2–8 months, and only 1–4% of all patients with pancreatic adenocarcinoma survive 5 years after diagnosis (Singh *et al*, 2004). According to an estimate of the American Cancer Society, 42 470 Americans were diagnosed with pancreatic cancer in 2009 and 35 240 died from it, marking this malignancy as the fourth leading cause of cancer deaths (Jemal *et al*, 2009). Surgical resection is the best and most effective choice for treatment, but in majority of the cases, the disease is locally advanced or has already metastasised to distant organs at the time of diagnosis. In the latter scenario, chemotherapy is considered as an option, but the effects are usually modest due to chemoresistance (Liau and Whang, 2008; Rejiba *et al*, 2009). Drug resistance in pancreatic cancer cells is thought to occur mainly as a result of active survival mechanisms and/or inefficient drug delivery because of the fibrotic nature of pancreatic tumours (Olive *et al*, 2009; Pei *et al*, 2009). Hence, there is an urgent need to develop alternative strategies and novel therapeutics for effective treatments of this devastating malignancy and improve clinical outcome.

The chemokine receptor, CXCR4, is expressed in a variety of malignancies and has been extensively studied for its role in cancer pathogenesis (Singh *et al*, 2007; Gelmini *et al*, 2008). CXCR4 expression is elevated in majority of pancreatic cancer tissues and precancerous lesions, suggesting its role in pancreatic cancer pathogenesis (Thomas *et al*, 2008; Marechal *et al*, 2009). CXCL12, a ligand for CXCR4, is also abundantly produced by neighbouring stromal cells and activation of CXCR4-expressing pancreatic cancer cells by CXCL12 leads to enhanced chemotaxis, transendothelial migration, and Matrigel invasion (Marchesi *et al*, 2004; Matsuo *et al*, 2009). Furthermore, high concentrations of CXCL12 are present at the common sites of pancreatic metastases (lymph nodes, liver, lungs, and so on), suggesting that CXCL12–CXCR4 signalling may have a role in the homing of pancreatic cancer cells to specific organs (Mori *et al*, 2004; Saur *et al*, 2005). Importantly, a recent study also showed that a distinct subpopulation of CD133^+^;CXCR4^+^ cancer stem cells (CSCs) was present at the leading edge of invasive pancreatic tumours, indicating a potential role of CXCR4 in the invasion process (Hermann *et al*, 2007). CXCR4 expressed on pancreatic CSCs was shown to be essential for their invasive and metastatic properties, suggesting a strong correlation with disease aggression (Hermann *et al*, 2007). The CXCL12–CXCR4 signalling axis has also been implicated in desmoplastic alterations of surrounding stroma favoring tumour cell growth (Marlow *et al*, 2008). In other studies, CXCL12–CXCR4 signalling was shown to stimulate pancreatic cancer cell proliferation and protection of cancer cells from serum deprivation-induced apoptosis (Koshiba *et al*, 2000; Marchesi *et al*, 2004; Saur *et al*, 2005; Marlow *et al*, 2008). Altogether, these observations indicate an important role of CXCR4 signalling in pancreatic cancer survival, proliferation, invasion, and metastasis, suggesting this signalling axis as a potential target for cancer therapy.

Gemcitabine is the only FDA-approved chemotherapeutic drug for the treatment of advanced and metastatic pancreatic cancer. However, it has not proven very effective clinically, and improvement in patient's survival undergoing gemcitabine therapy is only minimal (Olive *et al*, 2009; Wong and Lemoine, 2009). In this study, we hypothesised that the CXCL12–CXCR4 signalling axis is involved in pancreatic cancer drug resistance by stimulating intrinsic cell survival mechanisms. We have investigated the effect of CXCL12 in restricting the gemcitabine-induced toxicity of pancreatic cancer cells and activation of survival signalling pathways. Furthermore, we examined the therapeutic significance of a CXCR4 antagonist, AMD3100, in preventing the rescue effect of activated CXCL12–CXCR4 signalling. Our data demonstrate that CXCL12 induces a series of signalling events in pancreatic cancer cells and counteracts the cytotoxic effects of gemcitabine. In addition, our data show that AMD3100 can abrogate the survival effect of CXCL12–CXCR4 signalling and can serve as a therapeutic modality either alone or in combination with gemcitabine to effectively inhibit the growth of pancreatic cancer cells.

## Materials and Methods

### Cell lines and culture conditions

Human pancreatic cancer cell lines (Colo357, SW1990, AsPc1, BxPc3, CaPan1, HPAF II, CFPAC1, Panc1, MiaPaCa, Panc10. 05, Panc03.27, Panc02.03) were purchased from the American Type Culture Collection (Manassas, VA, USA). The cell lines were maintained in culture as adherent monolayer in RPMI-1640 or Dulbecco's Modified Eagle's Medium (DMEM) (Thermo Scientific, Logan, UT, USA) supplemented with 10% fetal bovine serum (FBS) (Atlanta Biologicals, Lawrenceville, GA, USA) and 100 *μ*M each of penicillin and streptomycin (Invitrogen, Carlsbad, CA, USA). Cells were grown at 37°C with 5% CO_2_ in humidified atmosphere.

### Reagents

SuperScript II Reverse Transcriptase and Vybrant MTT cell proliferation assay kit were from Invitrogen. Recombinant human CXCL12 and CXCL12 ELISA kit were purchased from R&D Systems (Minneapolis, MN, USA). AMD3100 octahydrochloride and anti-*β*-actin mouse monoclonal antibody were purchased from Sigma-Aldrich (St Louis, MO, USA). Gemcitabine was provided by USAMCI pharmacy. Phosphatase and protease inhibitors and FuGENE transfection reagent were from Roche Diagnostics (Mannheim, Germany). Antibody against CXCR4 (rabbit polyclonal) was from Abcam (Cambridge, MA, USA). Anti-Akt, -pAkt, and -pFAK (rabbit monoclonal) antibodies were from Epitomics (Burlingame, CA, USA). Antibodies for ERK1/2 (rabbit monoclonal), pERK1/2 (mouse monoclonal), Bcl-2 (rabbit polyclonal), BAD (rabbit monoclonal), pBAD (rabbit polyclonal), Bcl-xL (rabbit monoclonal), focal adhesion kinase (FAK) (rabbit polyclonal), and Survivin (rabbit monoclonal) were from Cell Signaling Technologies (Beverly, MA, USA). The Notch1 (goat polyclonal) and secondary horseradish peroxidase-conjugated anti-rabbit, anti-mouse, and anti-goat antibodies were purchased from Santa Cruz Biotechnology (Santa Cruz, CA, USA). DNAzol reagent was from Molecular Research (Cincinnati, OH, USA). CaspACE FITC-VAD-FMK and Dual Luciferase Assay System kit were from Promega (Madison, WI, USA). ECL Plus Western Blotting detection kit, DharmaFECT transfection reagent, ON-TARGET *plus* non-targeting pool scrambled siRNAs and SMARTpool siRNAs targeting CXCR4 were from Thermo Scientific. LY294002 and PD98059 (PI3K and MEK1 inhibitors, respectively) were purchased from Cell Signaling Technology. TOPflash or FOPflash reporter plasmids were kindly provided by Dr R Samant, USAMCI, and pGL4.32[*luc*2*P*/NF-kB-RE/Hygro] reporter plasmid was purchased from Promega.

### Western blot analysis

Cells were processed for protein extraction and western blotting using standard procedures. Briefly, the cells were washed twice with phosphate-buffer saline (PBS) and scraped into NP-40 lysis buffer containing protease and phosphotase inhibitors. Cell lysates were passed through a needle syringe to facilitate the disruption of the cell membranes and were centrifuged at 14 000 r.p.m. for 20 min at 4°C, and supernatants were collected. Proteins (10–50 *μ*g) were resolved by electrophoresis on 10% SDS–PAGE, transferred onto polyvinylidene difluoride membrane and subjected to standard immunodetection procedure using specific antibodies: Akt, pAkt ERK1/2 pERK1/2, Bcl-2, Bcl-xL, FAK, pFAK, Survivin, *β*-catenin and BAD (1 : 1000), pBAD (1 : 500), Notch-1 (1 : 200), and *β*-actin (1 : 20 000). All secondary antibodies were used at 1 : 2500 dilutions. Blots were processed with ECL Plus Western Blotting detection kit and the signal detected using an LAS-3000 image analyzer (Fuji Photo Film Co., Tokyo, Japan).

### Enzyme-linked immunosorbent assay

Cells (1 × 10^6^) were seeded in six-well plates containing growth medium supplemented with FBS and cultured overnight. After 24 h, growth media were removed, and cells were conditioned in serum-free medium for next 72 h. The culture media were then collected, centrifuged at 1500 r.p.m. for 5 min to remove particles, and supernatants frozen at −80°C until use. CXCL12 was measured using an ELISA kit according to the manufacturer's instructions.

### LEF/TCF and NF-*κ*B transcriptional activity assays

To measure the LEF/TCF and NF-*κ*B transcriptional activity, pancreatic cancer cells (1 × 10^5^) were seeded in 12-well plates. After 24 h incubation, cells were transiently transfected with the luciferase promoter-reporter constructs (TOPflash, FOPflash, or pGL4.32[*luc*2*P*/NF-kB-RE/Hygro]). TOPflash reporter plasmid contains three copies of the Tcf/Lef sites upstream of a thymidine kinase (TK) promoter and the Firefly luciferase gene, whereas in FOPflash, Tcf/Lef sites are mutated and therefore it serves as a control for measuring nonspecific activation of the reporter. Cells were also co-transfected with a reporter plasmid, containing Renilla reniformis luciferase gene downstream of the TK promoter, to control for the transfection efficiency. All transfections were performed using FuGENE as a transfection reagent according to the manufacturers’ recommendations. Cells were treated with CXCL12 (100 ng ml^−1^) 24 h post-TOPflash/FOPflash or pGL4.32[*luc*2*P*/NF-kB-RE/Hygro] transfection, and after the next 24 h, total protein was isolated in passive lysis buffer. Luciferase activity was measured using the Dual Luciferase Assay System. All experiments were carried out in triplicate and relative luciferase activity reported as the fold induction after normalisation for transfection efficiency.

### Cell viability assay

Panc1 and MiaPaCa cells were seeded in 96-well plates at a density of 5000 cells per well, followed by next day treatment with increasing concentration of gemcitabine (0–10 *μ*M) in the presence or absence of CXCL12 (100 ng ml^−1^). After 72 h of treatment, cell growth was determined by using Vybrant MTT cell proliferation assay. Growth was calculated as percent (%)=[((*A*/*B*)−1) × 100], where *A* and *B* are the absorbance of treatment and control cells, respectively. To examine the effect of CXCR4 targeting, cells were preincubated with small-molecule CXCR4 antagonist, AMD3100 (5 *μ*g ml^−1^), for 1 h. To delineate the role of Akt and ERK pathways, cells were pretreated for 1 h with LY294002 (20 *μ*M) and PD98059 (25 *μ*M), respectively. To further corroborate the role of CXCR4, Panc1 and MiaPaCa cells were also transiently transfected with CXCR4- or non-targeted siRNA pools using DharmaFECT transfection reagent (Thermo Scientific) as per the manufacturer's protocol. After 24 h, cells were treated with gemcitabine (5 *μ*M) and cells viability was examined 72 h post-treatment.

### DNA fragmentation assay

Panc1 and MiaPaCa cells were cultured in 10% DMEM with and without gemcitabine (5 and 10 *μ*M) and CXCL12 (100 ng ml^−1^) for 48 h. Cells were washed twice with PBS and DNA (2 *μ*g) was extracted using DNAzol reagent. Isolated DNA was resolved on 1.0% agarose gel containing ethidium bromide (EtBr) and observed under a LAS-3000 image analyzer (Fuji Photo Film Co.).

### Measurement of apoptosis *in situ*

Panc1 and MiaPaCa cells were cultured on chamber slides and treated with gemcitabine and/or CXCL12 as described previously. Apoptosis was detected by staining the cells with CaspACE FITC-VAD-FMK solution in PBS for 2 h at 37°C. CaspACE FITC-VAD-FMK *In Situ* Marker is a fluorescent analogue of the pancaspase inhibitor Z-VAD-FMK (carbobenzoxy-valyl-alanyl-aspartyl-[*O*-methyl]-fluoromethylketone), which irreversibly binds to activated caspases and is a surrogate for caspase activity *in situ*. Following staining with CaspACE FITC-VAD-FMK, cells were fixed with 4% paraformaldehyde at room temperature and washed with PBS. The bound fluorescent marker was detected under a Nikon Eclipse TE2000-U fluorescent microscope (Nikon Instruments Inc., Melville, NY, USA).

### Statistical analysis

Each experiment was performed at least three times, and all the values were expressed as mean±s.d. The differences between the groups were compared using student's *t*-tests. A *P*-value of ⩽0.05 was considered statistically significant.

## Results

### Expression of CXCR4 and CXCL12 in pancreatic cancer cells and their growth responsiveness to CXCL12 stimulation

**Figure 1:**
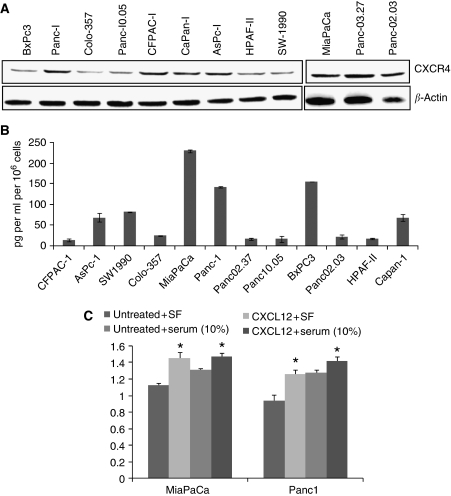
CXCR4 and CXCL12 expression and growth response in pancreatic cancer cells. (**A**) Total protein was isolated from 12 pancreatic cancer cell lines and resolved on 10% SDS–polyacrylamide gels by electrophoresis. Subsequently, the gels were immunoblotted with anti-CXCR4 rabbit polyclonal antibodies and reprobed with anti-*β*-actin (internal control) mouse monoclonal antibody. CXCR4 was expressed (at varying levels) in all pancreatic cancer cell lines tested. (**B**) Enzyme-linked immunosorbant assay (ELISA) was performed on used culture media from pancreatic cancer cells grown under serum-free condition for 72 h using a commercial kit. Low level CXCL12 expression (13–230 pg per ml per 10^6^ cells) was detected in all pancreatic cancer cell lines. (**C**) Growth response of pancreatic cancer cells (MiaPaCa and Panc1) on CXCL12 treatment (100 ng ml^−1^) indicating the functionality of CXCL12–CXCR4 signalling axis. CXCL12 stimulation (in serum-deprived and -supplemented media) led to the significant induction (^*^P<0.01) of growth in pancreatic cancer cells. Responses were more pronounced under serum-free conditions than in serum-containing cultures likely due to the compensatory growth-promoting effects of other serum factors.

Earlier it has been shown that CXCR4 is overexpressed in pancreatic tumour tissues and premalignant lesions (Thomas *et al*, 2008; Marechal *et al*, 2009). In addition, CXCR4 is also expressed by the pancreatic CSCs (Hermann *et al*, 2007). Here, we examined the expression of CXCR4 and its ligand CXCL12 by immunoblot and enzyme-linked immunosorbant assay (ELISA), respectively, in a panel of 12 pancreatic cancer cell lines. Our data show that all pancreatic cancer cell lines examined express CXCR4 and low levels of CXCL12 (13–230 pg per ml per 10^6^ cells) (Figure 1A and B). We further tested the growth responsiveness of pancreatic cancer cells to CXCL12 stimulation in two poorly differentiated pancreatic cancer cell lines, MiaPaCa and Panc1. The cells were treated with CXCL12 in serum-free and serum-containing culture media. In the absence of serum growth factors, CXCL12 stimulation led to the 29–33% induction of growth in pancreatic cancer cells, whereas a moderate increase (11–13%) was observed in the presence of serum growth factors (Figure 1C). These findings suggest that CXCL12–CXCR4 signalling is active in pancreatic cancer cells and can impact tumour cell growth.

### CXCR4 activation rescues pancreatic cancer cells from gemcitabine-induced cytotoxicity

**Figure 2:**
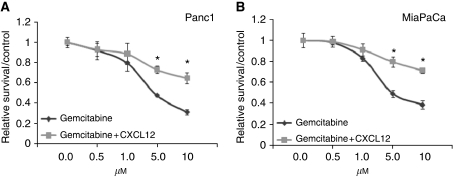
Rescue of pancreatic cancer cells from gemcitabine-induced toxicity on CXCL12 treatment. Two pancreatic cancer cell lines, Panc1 (**A**) and MiaPaCa (**B**), were treated with various doses of gemcitabine (0–10 *μ*M) under serum-supplemented condition in the presence and absence of CXCL12 (100 ng ml^−1^). Cancer cell viability was examined 72 h post-treatment by MTT assay. Significant protection of pancreatic cancer cells from gemcitabine toxicity (at 5 and 10 *μ*M) by CXCL12 was observed. Data are presented as relative survival with respect to untreated or CXCL12 only-treated cells to control for the growth-promoting effect of CXCL12 (^*^P<0.01).

**Figure 3:**
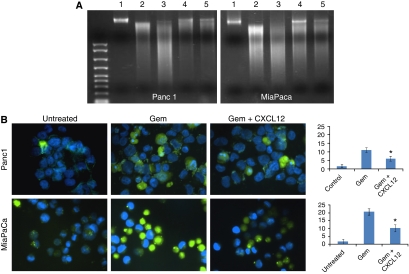
Antiapoptotic effects of CXCL12 treatment on gemcitabine-induced cell death. (**A**) DNA fragmentation assay. Cells were seeded in 6-cm Petri dishes and treated with 5 and 10 *μ*M gemcitabine in the absence or presence of CXCL12 (100 ng ml^−1^) for 48 h. Subsequently, genomic DNA was isolated and resolved (2 *μ*g per lane) on 1% agarose gel. Lane 1: untreated, lanes 2 and 3: gemcitabine-treated (5 and 10 *μ*M), respectively, and lanes 4 and 5: gemcitabine-treated (5 and 10 *μ*M, respectively) in the presence of CXCL12. CXCL12-treated pancreatic cancer cells exhibit reduced DNA laddering compared with cells treated with gemcitabine only. (**B**) *In situ* determination of apoptosis. Panc1 and MiaPaCa cells were cultured on chamber slides and treated with gemcitabine (5 *μ*M) in the absence and presence of CXCL12 (100 ng ml^-1^). Apoptosis was detected by staining the cells with CaspACE FITC-VAD-FMK solution in PBS for 2 h at 37°C. Following fixation, bound marker was visualised by fluorescent detection under a confocal microscope. Representative pictures (overlay of FITC and DAPI) are from one of the random fields of untreated, gemcitabine only, and gemcitabine+CXCL12-treated Panc1 and MiaPaCa cells. Apoptotic cells that stained positively with FITC-labelled marker were counted in 10 random fields and presented in a bar diagram (mean±s.d.). ^*^Significant difference as compared with gemcitabine only-treated cells. CXCL12 co-treated cells exhibited 53 and 55% reduced apoptosis by gemcitabine in Panc1 and MiaPaCa cells, respectively.

Although our data indicated minimal expression of CXCL12 by pancreatic cancer cells (Figure 1B), it is reported to be expressed at high levels by stromal cells and at sites of pancreatic cancer metastasis (Mori *et al*, 2004; Saur *et al*, 2005; Matsuo *et al*, 2009). Therefore, CXCL12–CXCR4 signalling might act in paracrine manner to influence pancreatic tumour growth and other malignant properties. In view of the fact that pancreatic cancer cells are highly resistant to chemotherapy, and gemcitabine (an FDA-approved drug) is only minimally effective against this malignancy, we investigated the role of CXCL12–CXCR4 signalling axis in pancreatic cancer chemoresistance. We treated pancreatic cancer cells (Panc1 and MiaPaCa) with various doses of gemcitabine (0–10 *μ*M) in the presence and absence of CXCL12 (100 ng ml^−1^) in serum-containing media. Our data show that CXCL12 treatment induced significant resistance (*P*<0.01) to gemcitabine cytotoxicity in both pancreatic cancer cell lines tested (Figure 2A and B). At 5.0 *μ*M dose of gemcitabine, we observed 52.3 and 50.7% cytotoxicity in Panc1 and MiaPaCa cells, respectively, as compared with untreated cells. In contrast, only 27.1 and 20.5% gemcitabine cytotoxicity, respectively, was reported in cells co-treated with CXCL12, indicating a significant survival advantage. To substantiate the role of CXCR4 in CXCL12-induced chemopreventive effect, Panc1 and MiaPaCa cells were transiently transfected with CXCR4- or non-targeted siRNA pools 24 h before gemcitabine treatment in the presence and absence of CXCL12. Resulting cell viability data show that CXCL12-induced cytoprotective effect is abolished when the cells are silenced for CXCR4 expression (Supplementary Figure S1). Next, we examined whether CXCL12-induced chemoresistance was due to its antiapoptotic effects on pancreatic cancer cells, DNA fragmentation and decreased caspase. Our data demonstrate that CXCL12-treated cells have reduced DNA fragmentation (Figure 3A) and decreased activity of caspases (Figure 3B) compared with cells treated with gemcitabine alone. These findings strongly suggest that CXCL12 treatment prevents apoptosis of pancreatic cancer cells by gemcitabine and suggest the implication of CXCL12-elicited survival pathways.

### CXCL12 treatment leads to FAK, Akt, and ERK activation

**Figure 4:**
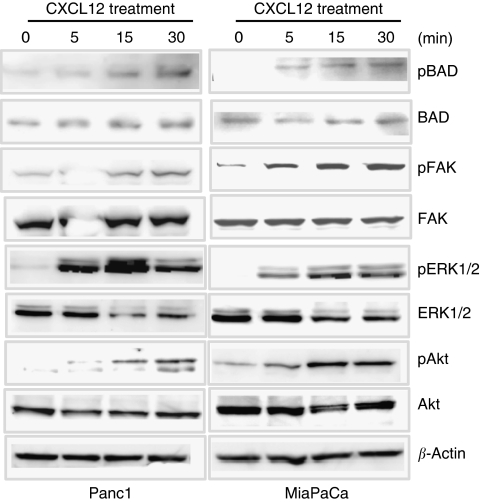
CXCL12-induced activation of FAK, Akt, and ERK pathways. Sub-confluent Panc1 and MiaPaCa cell cultures were treated with CXCL12 (100 ng ml^−1^) for 5, 15, and 30 min durations. Protein was extracted and resolved on SDS–polyacrylamide gels by electrophoresis. Activation of FAK, Akt, and ERK pathways was assessed by immunoblotting using total and phospho-form-specific antibodies as indicated. *β*-Actin served as an internal control. CXCL12 treatment induced the phosphorylation of all three effector proteins with a concomitant inactivating phosphorylation of proapoptotic BAD protein in both Panc 1 and MiaPaCa cell lines.

In next set of experiments, we examined the potential survival signalling pathways that might mediate the CXCL12-elicited chemoresistance. Because G-protein-coupled receptors transduce signals through diverse signalling pathways, including activation of FAK, PI3K/Akt, and ERK (Rozengurt, 2007), we investigated the activation of these signalling molecules in response to CXCL12 treatment. Pancreatic cancer cells (Panc1 and MiaPaCa) were briefly treated with CXCL12 (5–30 min), and activation of FAK, Akt, and ERK was examined by immunoprobing of total protein with phospho-form-specific antibodies. Our data revealed significant activation of all the three effector proteins in response to CXCL12 treatment (Figure 4). Both Akt and ERK have been shown to promote survival by phosphorylating BAD (a proapoptotic member of the Bcl-2 family) and thereby controlling its association with Bcl-xL or Bcl-2 (antiapoptotic members of the family) (Datta *et al*, 1997; Scheid and Duronio, 1998; Sheridan *et al*, 2008). Therefore, we examined the change in BAD phosphorylation in CXCL12-treated pancreatic cancer cells. Our data showed an increased level of phospho-BAD in both Panc1 and MiaPaCa cell lines treated with CXCL12 (Figure 4), suggesting that it could be one of the mechanisms by which CXCL12–CXCR4 signalling axis protects the pancreatic cancer cells from apoptosis.

### Enhanced transcriptional activities of *β*-catenin and NF-*κ*B and induction of survival proteins by CXCL12 treatment of pancreatic cancer cells

**Figure 5:**
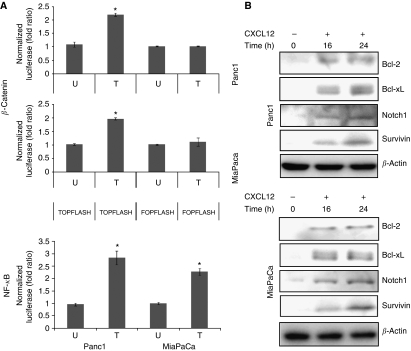
Induction of *β*-catenin/TCF and NF-*κ*B transcriptional activities and expression of survival proteins by CXCL12 in pancreatic cancer cells. (**A**) Pancreatic cancer cells were transfected with TOPflash or FOPflash or NF-*κ*B luciferase reporter constructs along with Renilla luciferase construct to control for the transfection efficiency. Cells were treated with CXCL12 24 h post-transfection and protein isolated in passive lysis buffer. Luciferase activity was assessed using a dual-luciferase assay system and data presented as fold change in luciferase activity after normalisation. Bars represent the average of triplicates±s.d.; ^*^statistically significant difference (*P*<0.01). (**B**) Change in the expression of Bcl-2, Bcl-xL, Notch 1, and survivin was examined in CXCL12-treated cells at different time durations by immunoblotting. An increased expression of all the four survival proteins was detected in CXCL12-treated pancreatic cancer cells.

In addition to directly influencing apoptotic signalling by BAD phosphorylation, both Akt and ERK can have indirect impacts on cell survival. The indirect routes involve the activation of *β*-catenin and NF-*κ*B that can elicit the expression of survival proteins. Therefore, we examined the transcriptional activities of *β*-catenin and NF-*κ*B responsive promoters after CXCL12 treatment in pancreatic cancer cells. Luciferase reporter assays indicated modest induction of transcriptional activity of *β*-catenin (2.05-fold (Panc1) and 1.92-fold (MiaPaCa)) and NF-*κ*B responsive promoter (2.98-fold (Panc1) and 2.26-fold (MiaPaCa)) in CXCL12-treated cells (Figure 5A). As activation of *β*-catenin and NF-*κ*B may culminate in the induction of important survival genes, we examined the expression of target prosurvival and antiapoptotic proteins. Our data showed that the expression of Bcl-2, Bcl-xL, Notch-1, and survivin proteins was significantly induced in response to CXCL12 treatment of pancreatic cancer cells (Figure 5B). These results suggest that the upregulation of key survival proteins may be another mechanism by which CXCL12–CXCR4 signalling axis protects the pancreatic cancer cells from gemcitabine-induced apoptotic cell death.

### Small-molecule CXCR4 antagonist, AMD3100, abrogates CXCL12-induced growth and gemcitabine resistance in pancreatic cancer cells

**Figure 6:**
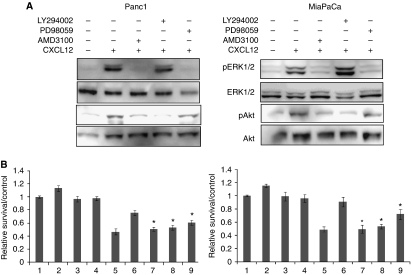
Effect of CXCR4 targeting and blockade of PI3K or Erk pathways on the cytoprotective effect of CXCL12 in pancreatic cancer cells from gemcitabine-induced toxicity. (**A**) Pancreatic cancer cells (Panc1 and MiaPaCa) were treated with AMD3100 (5 *μ*g ml^−1^) or LY294002 (20 *μ*M) or PD98059 (25 *μ*M) for 1 h before induction with CXCL12. Total protein was isolated 15 min after CXCL12 treatment, and activation of Akt and ERK was examined by immunoblotting for their total and phospho-forms. AMD3100 inhibited the activation of both Akt and ERK pathways, whereas LY294002 and PD98059 specifically inhibited Akt and ERK pathways, respectively. (**B**) Cells were pretreated with AMD3100 or LY294002 or PD98059 or PBS for 1 h. Subsequently, cells were treated with CXCL12 or gemcitabine either alone or in combination. Cell viability was assessed by MTT assay. Bars represent the average of triplicates±s.d.; ^*^statistically significant difference (*P*<0.01) with respect to gemcitabine+CXCL12-treated cells. Bars 1: untreated, 2: CXCL12 treated, 3: AMD3100 treated, 4: AMD3100 pretreated+CXCL12 treated, 5: gemcitabine treated, 6: gemcitabine+CXCL12 treated, 7: AMD3100 pretreated+gemcitabine+CXCL12 treated, 8: LY294002 pretreated+gemcitabine+CXCL12 treated, and 9: PD98059 pretreated+gemcitabine+CXCL12 treated.

Having observed a role of CXCR4 activation in gemcitabine resistance and potentiation of survival pathways, we investigated if the small-molecule CXCR4 antagonist, AMD3100, could diminish CXCL12-induced chemoresistance in pancreatic cancer cells. In addition, we also utilised pharmacological inhibitors of Akt (LY294002) and ERK (PD98059) signalling pathways, to delineate their role in the CXCL12-induced antiapoptotic response. Pancreatic cancer cells were treated for 1 h with AMD3100, LY294002, and PD98059 before treatment with CXCL12 alone or in combination with gemcitabine. Pretreatment with AMD3100 abolished the CXCL12-induced cell signalling, growth promotion, and chemoresistance of pancreatic cancer cells (Figure 6A and B). Although both the inhibition of Akt and ERK pathways had a significant negative impact on CXCL12-induced chemoresistance, a more potent effect of blockade of Akt signalling was observed (Figure 6B). These findings indicate that CXCL12-mediated survival response is signalled through the CXCR4 and mediated through the activation of Akt and ERK signalling pathways. This is particularly important considering the expression of a novel CXCL12 receptor, CXCR7, in pancreatic cancer cells at least at the transcript level (data not shown).

## Discussion

Pancreatic cancer, in most cases, is diagnosed late, when it has already advanced locally or metastasised to distant sites (Singh *et al*, 2004). Under this scenario, chemotherapy is the only treatment option. However, resistance to chemotherapy is a major clinical problem in pancreatic cancer and gemcitabine, the only FDA-approved drug for pancreatic cancer therapy improves the patients’ survival by only 2 weeks (Olive *et al*, 2009). Therefore, understanding the mechanisms of drug resistance in pancreatic cancer is a major focus in pancreatic cancer research to facilitate the development of novel therapeutic approaches or improve current therapy.

Chemokine signalling has long been implicated in cancer progression and metastasis through autocrine or paracrine mechanisms (Singh *et al*, 2007). Importantly, in previous studies, a chemokine receptor, CXCR4, was shown to be overexpressed in pancreatic cancer tissues and CSCs (Hermann *et al*, 2007; Thomas *et al*, 2008; Marechal *et al*, 2009) and has been shown to potentiate pancreatic cancer growth and invasion (Marchesi *et al*, 2004; Mori *et al*, 2004; Hermann *et al*, 2007). Here, we investigated another aspect of this signalling node in protecting pancreatic cancer cells from chemotherapeutic drug-induced apoptosis. We observed that all the pancreatic cancer cell lines tested expressed CXCR4, but low levels of CXCL12. Nonetheless, we observed significant protection of pancreatic cancer cells from gemcitabine toxicity on co-treatment with exogenous CXCL12, indicating a role for CXCL12–CXCR4 signalling axis in pancreatic cancer chemoresistance. As CXCL12 is abundantly expressed by stromal cells (Matsuo *et al*, 2009), this could be an exemplary example for the role of tumour microenvironment interaction in modulating the therapeutic response. To gain an insight into the mechanistic basis for the protective effects, we examined the activation of downstream signalling pathways. Consistent with previous reports (Glodek *et al*, 2007; Lu *et al*, 2009; Shen *et al*, 2010), CXCL12 induced the activation of FAK, Akt, and ERK. In a recent study, activation of FAK by extracellular matrix–integrin signalling was shown to promote the chemoresistance of pancreatic cancer cells (Huanwen *et al*, 2009). Akt and ERK have also been shown to promote survival signalling (Middleton *et al*, 2008), and constitutive or induced activation of ERK and Akt pathways has been previously associated with chemoresistant behaviour of pancreatic cancer cells (Yokoi and Fidler, 2004; Zhao *et al*, 2006). In fact, FAK-associated chemoresistance of pancreatic cancer cells was shown to be mediated, in part, by the activation of PI3K/Akt pathway (Huanwen *et al*, 2009).

Both Akt and ERK can transduce survival signals directly or indirectly. In direct course, Akt and ERK have been shown to phosphorylate BAD, a proapoptotic member of the Bcl-2 family (Datta *et al*, 1997; Scheid and Duronio, 1998; Sheridan *et al*, 2008). Phosphorylation prevents BAD from binding either Bcl-2 or Bcl-xL, and thus suppresses apoptosis. In the indirect route, induction of survival protein expression occurs through the activation of *β*-catenin and NF-*κ*B pathways. Activation of ERK has been shown to promote transactivation of *β*-catenin by phosphorylating *α*-catenin (Ji *et al*, 2009). Furthermore, Akt can activate *β*-catenin by inducing direct phosphorylation or by inactivating GSK-3*β* (Monick *et al*, 2001; Fang *et al*, 2007; Korkaya *et al*, 2009). In other reports, Akt pathway has been shown to regulate NF-*κ*B, and NF-*κ*B was shown to be essential for oncogenic transformation by PI3K and Akt (Ozes *et al*, 1999; Romashkova and Makarov, 1999; Sizemore *et al*, 1999; Madrid *et al*, 2000). Akt-induced activation of NF-*κ*B likely occurs through phosphorylation of IKK*α*, which then targets the I*κ*B inhibitor protein and phosphorylates the p65 NF-*κ*B subunit (Ozes *et al*, 1999; Madrid *et al*, 2000; Bai *et al*, 2009). Consistent with these findings, we also observed enhanced transcriptional activities of *β*-catenin and NF-*κ*B responsive promoters and expression of downstream targets in CXCL12-treated pancreatic cancer cells. Enhanced transcriptional activity of *β*-catenin and NF-*κ*B has been shown to induce epithelial to mesenchymal transition (EMT), and in recent studies, EMT has been associated with drug-resistant nature of pancreatic cancer cells (Li *et al*, 2009; Wang *et al*, 2009). In fact, relative drug-resistant nature of pancreatic cancer cells has been correlated with the mesenchymal phenotype (Shah *et al*, 2007). Other studies have shown that the underlying resistance to apoptosis is, in part, due to constitutive activation of NF-*κ*B in pancreatic cancer (Harikumar *et al*, 2010; Wang *et al*, 2010). Our results also indicate that the CXCL12-induced gemcitabine resistance in pancreatic cancer cells might, in part, also be due to the activation of NF-*κ*B and induction of downstream survival proteins (Bcl-2, Bcl-xL, survivin, and so on).

The use of small-molecule inhibitors represents an attractive targeted therapeutic approach. Previously, we showed that targeting of chemokine receptors, CXCR1 and CXCR2, in malignant melanoma by small-molecule antagonists led to reduced tumour growth, invasion, and angiogenesis (Singh *et al*, 2009). Here, we utilised AMD3100, a specific antagonist of CXCR4, to target CXCR4 activation in response to CXCL12 treatment and demonstrate its efficacy in abolishing the chemoprotective effect of CXCL12–CXCR4 signalling axis. The therapeutic potential of AMD3100 has been studied largely in fighting HIV infection (De, 2003), although there are also some recent reports that highlight its therapeutic use in cancer (Yasumoto *et al*, 2006; Azab *et al*, 2009). In the same line, our data also indicate that AMD3100 might be useful in targeting the CXCL12–CXCR4 signalling axis in pancreatic cancer. Pharmacokinetics and safety of AMD3100 has been studied in human volunteers after intravenous injection and shown to have minimal side effects (Hendrix *et al*, 2000). Therefore, AMD3100 can serve as a novel therapeutics for pancreatic cancer alone or in combination with cytotoxic drug.

In conclusion, our findings provide additional support for the pathological role of CXCL12–CXCR4 signalling in pancreatic cancer, and demonstrate, for the first time, a role for this axis in drug resistance. Our data show that the induced chemoresistance is partly mediated by the activation of Akt and ERK signalling pathways and a small-molecule antagonist against CXCR4 can effectively abrogate the survival signals and resensitise the pancreatic cancer cells to gemcitabine cytotoxicity. Therefore, future clinical trials in pancreatic cancer might benefit from targeting of this signalling axis alone or in combination with chemotherapy.
