# Supplementary material for: CXCL12–CXCR4 signalling axis confers gemcitabine resistance to pancreatic cancer cells: a novel target for therapy
Source: Br J Cancer. 2010 Nov 2;103(11):1671–9. doi: 10.1038/sj.bjc.6605968 (PMC2994230; doi:10.1038/sj.bjc.6605968)
Supplement: Supplementary Figure 1 [file 6605968x1.ppt]

## Slide 1
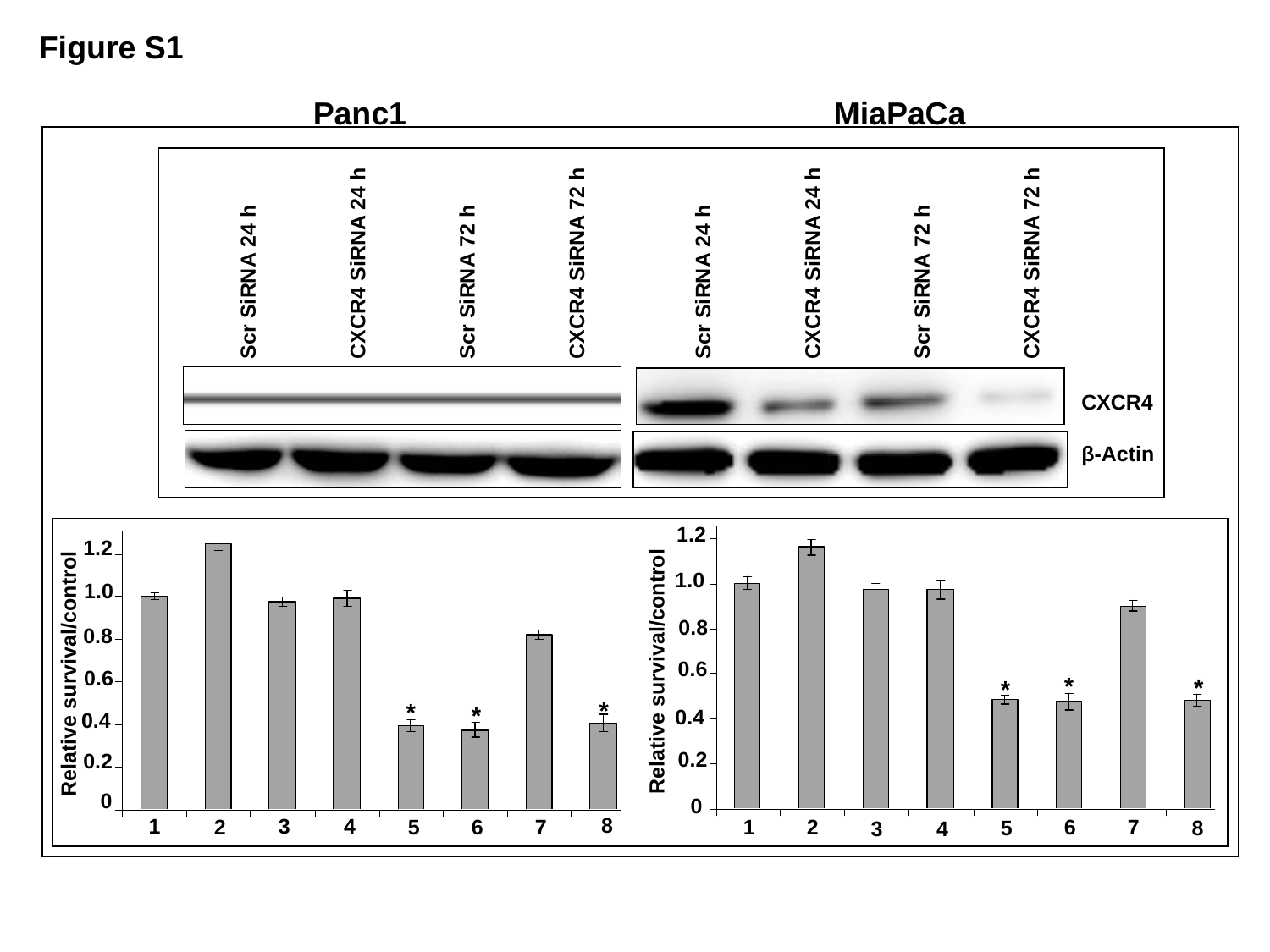

Figure S1
Panc1
MiaPaCa
Scr SiRNA 24 h
CXCR4 SiRNA 24 h
Scr SiRNA 72 h
CXCR4 SiRNA 72 h
Scr SiRNA 24 h
CXCR4 SiRNA 24 h
Scr SiRNA 72 h
CXCR4 SiRNA 72 h
CXCR4
β-Actin
1.2
1.2
1.0
1.0
0.8
0.8
0.6
Relative survival/control
Relative survival/control
0.6
*
*
*
*
*
*
0.4
0.4
0.2
0.2
0
0
8
1
3
4
2
6
7
5
1
2
6
7
5
8
3
4
