# Supplementary material for: CXCL12–CXCR4 signalling axis confers gemcitabine resistance to pancreatic cancer cells: a novel target for therapy
Source: Br J Cancer. 2010 Nov 2;103(11):1671–9. doi: 10.1038/sj.bjc.6605968 (PMC2994230; doi:10.1038/sj.bjc.6605968)
Supplement: Supplementary Figure legend [file 6605968x2.doc]

**Figure S1. Effect of CXCR4 silencing on the chemoprotective effect of CXCL12 in pancreatic cancer cells from gemcitabine-induced toxicity.** A. Pancreatic cancer cells (Panc1 and MiaPaCa) were transiently transfected with CXCR4- or non-targeted scrambled (Scr) siRNAs. Total protein was isolated at 24 and 72 h post-transfection and CXCR4 expression was examined by immunoblotting. Expression of CXCR4 was consistently decreased (≥ 70%) in both the cell lines at 24 and 72 h post-siRNA transfection. B. To analyze the effect of CXCR4 silencing on drug cytotoxicity, Panc1 and MiaPaCa cells were transiently transfected with CXCR4- or Scr siRNAs. After 24 h of transfection, cells were treated with CXCL12 and/or Gemcitabine. Cell viability was assessed by MTT assay. Bars represent the average of triplicates ± S.D.; *, statistically significant difference (p<0.01) with respect to Scr siRNA-pretreated + Gemcitabine + CXCL12-treated cells. Bars 1: Scr siRNA- pretreated, 2: Scr siRNA-pretreated + CXCL12-treated, 3: CXCR4 siRNA- pretreated, 4: CXCR4 siRNA-pretreated + CXCL12-treated, 5: Scr siRNA-pretreated + gemcitabine-treated, 6: CXCR4 siRNA-pretreated + gemcitabine-treated, 7: Scr siRNA- pretreated + gemcitabine + CXCL12-treated, and 8: CXCR4 siRNA-pretreated + gemcitabine + CXCL12-treated.
